# Erythrocytic α-Synuclein Species for Parkinson’s Disease Diagnosis and the Correlations With Clinical Characteristics

**DOI:** 10.3389/fnagi.2022.827493

**Published:** 2022-02-03

**Authors:** Zhenwei Yu, Genliang Liu, Yang Li, Ehsan Arkin, Yuanchu Zheng, Tao Feng

**Affiliations:** ^1^Beijing Neurosurgical Institute, Capital Medical University, Beijing, China; ^2^Center for Movement Disorders, Department of Neurology, Beijing Tiantan Hospital, Capital Medical University, Beijing, China; ^3^China National Clinical Research Center for Neurological Diseases, Beijing, China; ^4^Institute of Systems Biomedicine, School of Basic Medical Sciences, Peking University Health Science Center, Beijing, China

**Keywords:** α-syn, erythrocyte, Parkinson’s disease, electrochemiluminescence, depression, anxiety

## Abstract

**Background:**

Erythrocytes contain most of the peripheral α-synuclein (α-syn), which is the key pathological molecular of α-synucleinopathies including Parkinson’s disease (PD). Our objectives were to assess the efficiency of erythrocytic total and oligomeric α-syn levels as PD diagnostic biomarkers, and to identify the correlations between erythrocytic α-syn levels and physiological/psychiatrical assessment scales.

**Methods:**

Home-brewed electrochemiluminescence assays were applied to assess the concentrations of erythrocytic total and oligomeric α-syn levels in a cohort including 124 patients with PD and 79 healthy controls (HCs). The correlations between erythrocytic α-syn levels and clinical measurements were assessed using Spearman’s rank test.

**Results:**

Both the erythrocytic total and oligomeric α-syn levels were significantly higher in PD patients than HCs. The biomarkers adjusted for age and sex discriminated PDs from HCs well with 80% sensitivity, 89% specificity and 79% sensitivity, 83% specificity, respectively. Combining erythrocytic total and oligomeric α-syn levels by using binary logistic regression analysis with the controlling of age and sex generated a factor discriminates PDs from HCs with 88% sensitivity and 85% specificity. The erythrocytic total but not oligomeric α-syn levels adjusted for age and sex significantly correlated with anxiety scales and the MDS-UPDRS III scales in PD patients, respectively.

**Conclusion:**

We showed the usefulness of erythrocytic total and oligomeric α-syn levels as biomarkers for PD. Our results also suggest the capability of erythrocytic α-syn as a potential pathological factor and therapeutic target for psychiatric symptoms in PD patients.

## Introduction

Parkinson’s disease (PD) is one of the most common neurodegenerative diseases, featured by neuronal α-synuclein (α-syn) pathological aggregation and the loss of dopaminergic neurons in the midbrain. Currently, the diagnosis of PD is mainly based on clinical symptoms, which leads to a varying diagnostic accuracy according to clinical expertise and disease duration (Kalia and Lang, 2015). Cerebrospinal fluid (CSF) α-syn and it’s species were widely studied as potential biomarkers for PD diagnosis (Tokuda et al., 2010; Parnetti et al., 2019). However, despite the blood derived α-syn contamination in CSF, which is commonly seen (Hong et al., 2010), routinely collection of CSF is difficult in many ways for patients with neurodegenerative diseases and healthy controls (HCs) due to the invasive procedures.

Plasma and erythrocytes are more readily available sources of biomarkers. In recent years, it has been found that more than 99% of peripheral α-syn is derived from erythrocytes (Barbour et al., 2008). Although the role α-syn plays in erythrocyte is blurred, studies have reported that aggregated α-syn was found higher on the membranes of erythrocytes from PD patients than HCs (Tian et al., 2019). Furthermore, altered morphology of erythrocytes was noticed in PD patients (Pretorius et al., 2014). These results suggest a possible correlation of erythrocytic α-syn with PD pathology and clinical characteristics.

Although PD mainly manifests motor abnormality, a series of non-motor symptoms including depression and anxiety were also commonly seen in patients with PD (Reijnders et al., 2008; Broen et al., 2016). Beyond the motor features, psychiatric symptoms seriously affect the life quality of PD patients and even their families, which requires further attention (Schrag, 2006; Upneja et al., 2021). Studies have demonstrated that PD related pathological changes occur in both motor and non-motor regions of brain including substantia nigra (SN), cortex, olfactory bulb, etc., which reflect the autonomic dysfunctions in PD (Maillet et al., 2016). However, the understanding of pathogenesis of psychiatric symptoms in PD is very limited. The purpose of this study is to learn the performance of erythrocytic α-syn in PD diagnosis and the correlation between erythrocytic total/oligomeric α-syn and PD clinical symptoms including motor function and depression/anxiety scales.

## Materials and Methods

### Demographic and Clinical Characteristics

The demographic information and clinical measurements of 124 PD patients and 79 healthy controls are presented in Table 1. All participants were recruited from Beijing Tiantan Hospital, and the PD diagnostic criteria were in accordance with those of the Parkinson’s UK Brain Bank and the Movement Disorder Society (MDS) Clinical Diagnostic Criteria for PD (Postuma et al., 2015). Control subjects without neurological diseases were recruited from the Tiantan Hospital physical examination center. Exclusion criteria were as follows: (1) atypical or secondary parkinsonian syndromes; (2) a history of stroke, moderate to severe head trauma, hydrocephalus, brain tumor, or deep brain stimulation implantation. This study was approved by the Institutional Review Board, and written informed consent was obtained from all participants before inclusion in the study. The disease severity was assessed using Movement Disorder Society sponsored Unified Parkinson’s Disease Rating Scale Part-III (MDS-UPDRS III) rating and Hoehn and Yahr (H&Y) staging. Montreal Cognitive Assessment (MoCA) and Mini-mental State Examination (MMSE) were used to detect the cognitive functioning. The depression and anxiety scores were assessed using the 17-item Hamilton Depression Rating Scale (HAMD) and Hamilton Anxiety Rating Scale (HAMA).

**TABLE 1:** Demographic information, clinical characteristics, and erythrocytic biomarker levels.

	HC	PD	Significance
Number of subjects	79	124	
Age (mean ± SD)	62.6 ± 7.9	61.2 ± 8.5	0.664^1^
Sex (men: women)	44: 35	67: 57	0.816^2^
MDS-UPDRS III (median, range, *N* = 74)	NA	40.0 (8–73)	NA
HAMA (median, range, *N* = 70)	NA	14 (3–47)	NA
HAMD (median, range, *N* = 70)	NA	14 (2–54)	NA
H&Y (median, range, *N* = 90)	NA	3 (1–5)	NA
MMSE (median, range, *N* = 67)	NA	27 (16–30)	NA
MoCA (median, range, *N* = 67)	NA	22.5 (6–29)	NA
Erythrocytic α-syn (mean ± SD, ng/mL)	645.0 ± 248.0	1520.5 ± 824.5	**<0.001^3^**
Erythrocytic oligomeric α-syn (mean ± SD, ng/mL)	135.0 ± 42.2	218.1 ± 86.5	**<0.001^3^**

*^1^Based on Mann–Whitney test.*

*^2^Based on Chi-square test.*

*^3^Based on univariate general linear model with the controlling of age and sex.*

*All significant p-values are highlighted by bold characters.*

*HC, healthy control; PD, Parkinson’s disease; MDS-UPDRS III, Movement Disorder Society sponsored Unified Parkinson’s Disease Rating Scale Part-III; α-syn, α-synuclein.*

### Blood and Erythrocyte Sample Preparations

Venous blood samples were collected in the morning using polypropylene collection and storage tubes with EDTA (BD Biosciences, CA, United States). The samples were centrifuged at 4°C and 2,000 × *g* for 15 min within 1 h since collection, and the erythrocytes were in the lower layer. 2 μl erythrocytes were mixed with 198 μl pre-chilled STET buffer (pH8.0 Leagene, Beijing, China) for protein extraction. The mixtures were vortexed for 15 s and rotated at 4°C for 30 min, followed by centrifugation at 4°C 12,000 × *g* for 10 min in order to get rid of the cell debris. The supernatant was transferred into a new tube and stored at −80°C for further analysis.

### Meso Scale Discovery Electrochemiluminescence Immunoassays

Home-brewed 96-well Meso Scale Discovery (MSD, Rockville, MD, United States) U-Plex plates were used for the quantification of erythrocytic total and oligomeric α-syn. Recombinant α-syn monomers (Alpha-synuclein Protein – monomer; Cat# PR-001, Proteos, Inc., Kalamazoo, MI, United States) and filaments (Alpha-synuclein Protein – filament; Cat# PR-002, Proteos, Inc.) were used as standard proteins. The Sulfo-TAG labeled anti-α-syn clone 42 (610786, BD Bioscience, CA, United States) was used as detector for both total and oligomeric α-syn assays. The assays were described as reported previously (Tian et al., 2019). Briefly, capture antibodies for total α-syn (ab138501, Abcam, Cambridge, MA, United States) and α-syn filaments (ab209538, Abcam, Cambridge, MA, United States) were biotinylated and coated on the MSD U-Plex plates. Excess capture antibodies were eluted three times with 150 μl 1× wash buffer (MSD, Rockville, MD, United States), and the plates were blocked using 150 μl Diluent 35 (D35, MSD, Rockville, MD, United States) for 1 h with 600 rpm shaking. Samples were diluted (1:100 dilution in D35 for total α-syn measurement and raw STET lysis for α-syn filament measurement) and loaded together with standard proteins to the pre-coated plates for 1 h with 600 rpm shaking. After three times of washing, 50 μl Sulfo-TAG-labeled detection antibody solution at the concentration of 1 μg/ml was applied for 1 h with 600 rpm shaking. Afterward, the detection antibody was washed off, and 150 μl 2 × Read Buffer T (MSD, Rockville, MD, United States) was applied for protein quantification in Sector Imager 6000 (MSD, Rockville, MD, United States). The concentrations were then normalized to the original erythrocyte volume.

### Statistical Analysis

IBM SPSS 25 (IBM, Chicago, IL, United States) and GraphPad Prism 9 (GraphPad Software, La Jolla, CA, United States) were used for statistical analyses. Univariate general linear model adjusted by age and sex was used to determine the differences of the biomarkers between PD and HC. Multivariable logistic regression model was used for differentiating PD from HC, controlling for age and sex of participants. The model included age, sex, erythrocytic total α-syn and oligomeric α-syn concentrations. Partial correlation analysis with the controlling of age and sex was used to assess the correlations of erythrocytic total or oligomeric α-syn concentrations with clinical characteristics. Receiver operating characteristic (ROC) curves were used to evaluate the sensitivities and specificities in distinguishing PD from healthy controls. The maximal sum of sensitivity and specificity was determined as the “optimum” cutoff value for a ROC curve. *P* < 0.05 was regarded as significant.

## Results

### Demographic Information, Clinical Characteristics, and Erythrocyte Measurements

**FIGURE 1:**
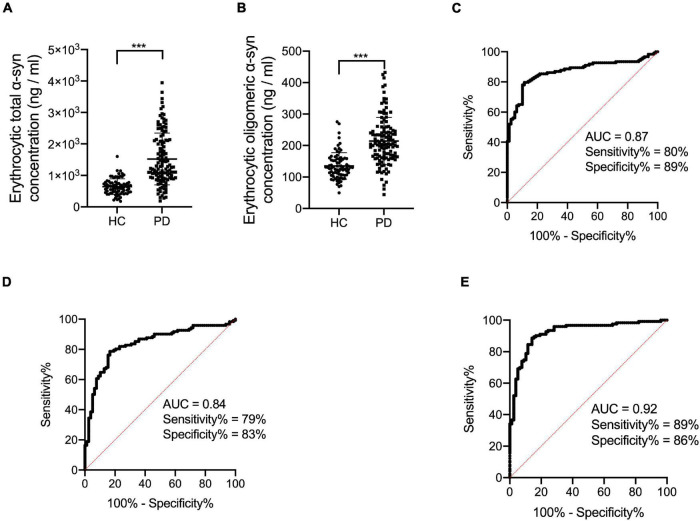
Evaluation of erythrocytic total and oligomeric α-syn concentrations and the receiver operating characteristic curves for erythrocytic α-syn species and an integrative model to differentiate PD from HC. **(A)** Assessment of erythrocytic total α-syn levels. ***p < 0.001. **(B)** Assessment of erythrocytic oligomeric α-syn levels. ***p < 0.001. **(C)** Receiver operating characteristic curve for erythrocytic total α-syn adjusted for age and sex to differentiate PD from HC. **(D)** Receiver operating characteristic curve for erythrocytic oligomeric α-syn adjusted for age and sex to differentiate PD from HC. **(E)** Receiver operating characteristic curve for the multivariable model to differentiate PD from HC. The multivariable model includes erythrocytic total α-syn and erythrocytic oligomeric α-syn levels, controlling for age and sex. Univariate general linear model with the controlling of age and sex was used for two factor comparisons. HC, healthy control; PD, Parkinson’s disease; AUC, area under curve; α-syn, α-synuclein.

The demographic information, clinical characteristics and erythrocyte measurements are summarized in Table 1. The HCs were age and sex ratio matched with PD subjects (Table 1). The levels of both erythrocytic total and oligomeric α-syn were significantly higher in PD subjects compared to healthy controls (Table 1 and Figures 1A,B, *p* < 0.001, *p* < 0.001). All clinical characteristics were listed in Table 1.

### Diagnostic Performance of Biomarkers

In order to analyze the diagnostic performance of the biomarkers for PD, ROC analysis was performed based on erythrocytic total and oligomeric α-syn concentrations adjusted for age and sex. The area under curve (AUC) of erythrocytic total α-syn for the diagnosis of PD is 0.87 (95% CI 0.82–0.92), with 80% sensitivity and 89% specificity (Figure 1C). For the erythrocytic oligomeric α-syn, the sensitivity and specificity are 79 and 83% for PD diagnosis, and the AUC is 0.84 (95% CI 0.78–0.90) (Figure 1D).

Then we performed a multivariable logistic regression including erythrocytic total and oligomeric α-syn concentrations as well as age and sex for discriminating PD patients. The AUC of this multivariable model is 0.92 (95% CI 0.88–0.96), with 89% sensitivity and 86% specificity (Figure 1E).

### Correlation Analysis of Clinical Characteristics and Erythrocytic α-Synuclein

**FIGURE 2:**
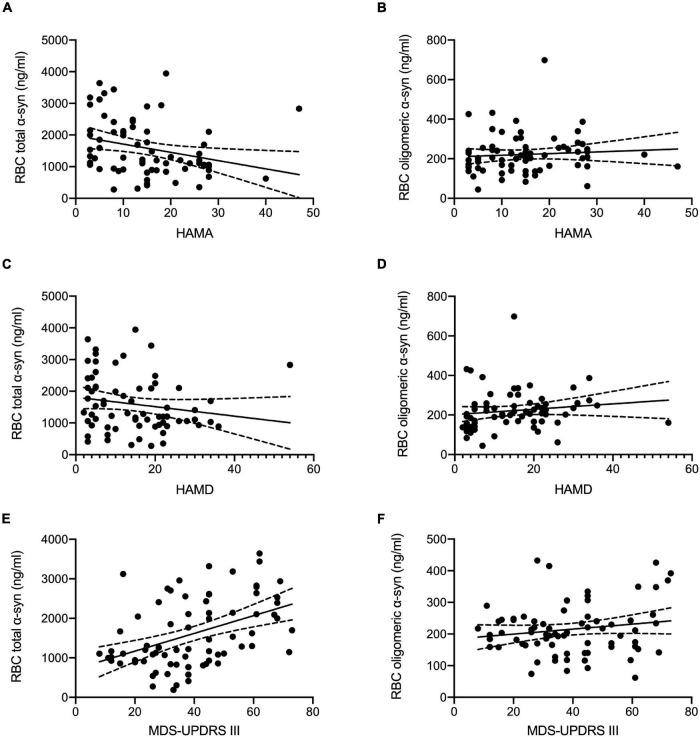
Correlation analysis of erythrocytic total and oligomeric α-syn concentrations with clinical characteristics in PD. **(A)** Erythrocytic total α-syn concentrations adjusted by age and sex were significantly correlated with HAMA scales (*p* = 0.016, *r* = –0.292) in PD patients. **(C)** No significant correlations between erythrocytic total α-syn concentrations and HAMD scales (*p* = 0.140, *r* = –0.182) were observed in PD patients. **(B,D)** Erythrocytic oligomeric α-syn concentrations were not correlated with HAMD scales (*p* = 0.291, *r* = 0.131) and HAMA scales (*p* = 0.649, *r* = 0.057) in PD patients. **(E)** Significant correlations between MDS-UPDRS III scores and erythrocytic total α-syn levels (*p* < 0.001, *r* = 0.455) were found in PD patients. **(F)** No correlations were found between MDS-UPDRS III scores and erythrocytic oligomeric α-syn levels (*p* = 0.368, *r* = 0.107). Dash lines represent 95% confidence intervals. Partial correlation analysis with the controlling of age and sex was used to assess the correlations. α-syn, α-synuclein; HAMA, Hamilton Anxiety Rating Scale; HAMD, 17-item Hamilton Depression Rating Scale; MDS-UPDRS III, Movement Disorder Society sponsored Unified Parkinson’s Disease Rating Scale Part-III; RBC, red blood cell.

The correlations between erythrocytic α-syn concentrations and clinical characteristics including MDS-UPDRS III, H&Y, HAMA, HAMD, MoCA, and MMSE in patients with PD were summarized in Table 2 and Figure 2. Briefly, erythrocytic total α-syn concentrations adjusted for age and sex were significantly associated with MDS-UPDRS III scores (Figure 2E, *p* < 0.001) and HAMA scores (Figure 2A, *p* = 0.016), but they were not associated with HAMD scores (Figure 2C, *p* = 0.140). The erythrocytic oligomeric α-syn concentrations adjusted for age and sex were neither correlated with HAMA and HAMD scores in PD patients (Figures 2B,D, *p* = 0.649, *p* = 0.291) nor MDS-UPDRS III scores (Figure 2F, *p* = 0.368).

**TABLE 2:** Correlation of erythrocytic biomarkers with clinical characteristics.

Erythrocytic biomarkers	Diagnosis		HAMA (*N* = 70)	HAMD (*N* = 70)	MDS-UPDRS III (*N* = 74)	H&Y (*N* = 90)	MMSE (*N* = 67)	MoCA (*N* = 67)
Total α-syn	PD	*r*	−0.292	−0.182	0.455	0.170	−0.134	−0.153
		*p*	**0.016**	0.140	**<0.001**	0.113	0.291	0.227
Oligomeric α-syn	PD	*r*	0.057	0.131	0.107	−0.106	−0.147	−0.152
		*p*	0.649	0.291	0.368	0.327	0.248	0.229

*Partial correlation analysis with the controlling of age and sex was used to assess the r and p -values between erythrocytic biomarkers and clinical characteristics.*

*PD, Parkinson’s disease; α-syn, α-synuclein; HAMA, Hamilton Anxiety Rating Scale; HAMD, 17-item Hamilton Depression Rating Scale; MDS-UPDRS III, Movement Disorder Society sponsored Unified Parkinson’s Disease Rating Scale Part-III; H&Y, Hoehn & and Yahr scale; MMSE, Mini-mental State Examination; MoCA, Montreal Cognitive Assessment.*

*All significant p-values are highlighted by bold characters.*

## Discussion

In the current study, we investigated the erythrocytic total/oligomeric α-syn concentrations in PD patients and healthy controls, as well as their correlations with clinical characteristics including MDS-UPDRS III, H&Y, MoCA, MMSE, HAMA, and HAMD with the controlling of age and sex. We found that erythrocytic total and oligomeric α-syn concentrations were significantly higher in PD patients than healthy controls. The results were consistent with several previous findings (Wang et al., 2015; Tian et al., 2019), suggesting the possibility of peripheral α-syn involvement in PD pathology. Tian et al. (2019) explored the total, aggregated and phosphorylated α-syn in cytoplasm and membrane of erythrocytes, and found both total and aggregated α-syn levels in erythrocytic membrane were significantly higher in PD patients. Interestingly, they didn’t see the differences of total and aggregated α-syn levels in erythrocyte whole lysates between PD patients and HCs. One major difference between the two analyses is that they normalized the erythrocytic total α-syn levels to total erythrocytic protein concentrations, and the aggregated α-syn levels to total α-syn concentrations, while we normalized both erythrocytic total and aggregated α-syn levels to the volume of erythrocytes. It has been reported PD patients’ oligomeric α-syn levels in CSF and plasma were higher than controls (El-Agnaf et al., 2006; Tokuda et al., 2010; Majbour et al., 2016; Eusebi et al., 2017). However, the origin of α-syn was not well studied. Our study suggests that erythrocytic α-syn is elected and may reflect the pathogenesis of PD in the periphery, which should be validated as a predictive biomarker in a prodromal cohort in the future.

We found erythrocytic total and oligomeric α-syn are potential PD diagnostic biomarkers yielding even higher sensitivity and specificity than the results based on CSF α-syn concentrations or previously published erythrocytic α-syn levels (Mollenhauer et al., 2008; Hong et al., 2010; Shi et al., 2011; Wang et al., 2015; Tian et al., 2019). Notably, our results were achieved from a relatively small cohort (*N* = 129 for PD patients). Cohort expansion will be needed for the validation of the diagnostic efficiency of these biomarkers for PD in the future. Additionally, our cohort doesn’t recruit patients with multiple system atrophy (MSA), which is another important α-synucleinopathy and commonly misdiagnosed as PD especially at disease early phase (Fanciulli et al., 2019). It is important to assess the α-synucleinopathy discriminative efficiency in the future in order to better utilize our biomarkers in clinical diagnosis.

Psychiatric symptoms including depression and anxiety are commonly seen in patients with PD, and badly affect the quality of life in PD patients and their families (Kano et al., 2011; Piredda et al., 2020). Most of the PD patients with depression also suffer from anxiety (Kano et al., 2011; Cui et al., 2017). Fan et al. (2016) found only 2.2% PD patients are suffering from depression alone in a cohort from Taiwan Region. It has been reported that several risk factors including poor sleep quality, tumor, not having partner, anxiety were associated with depression, and depression, autonomic dysfunction, larger SN area and rapid eye movement behavior disorder (RBD) were associated with anxiety in PD patients (Cui et al., 2017). Negre-Pages et al. (2010) found depression in PD patients was associated with reduced dopamine transporter (DAT) activity and motor dysfunction. However, it is still elusive if depression and anxiety were the psychological responses of progressive motor and non-motor disability. Caudal et al. (2015) found that α-syn overexpressing in dopaminergic neurons from midbrain induced a depressive-like phenotype in Sprague-Dawley rats. Another study based on late life major depressive disorder (MDD) didn’t found CSF α-syn levels altered between MDD and control participants (Bruno et al., 2021). Although α-syn level in peripheral blood is much higher than CSF and more than 99% of peripheral α-syn is from erythrocyte (Barbour et al., 2008; Hong et al., 2010; Shi et al., 2010), up-to-date, no study has explored the correlation of erythrocytic α-syn levels with depression and anxiety in PD patients.

In the current study, for the first time we discovered that the anxiety levels assessed by HAMA scales were negatively associated with erythrocytic total α-syn concentrations. Interestingly, we didn’t see the association of MDS-UPDRS III motor scales with HAMD/HAMA scales in PD patients. This result suggests that the depression and anxiety symptoms are not likely the secondary psychological responds of PD motor disability, and erythrocytic total/oligomeric α-syn is potential therapeutic target for those symptoms. However, one of the most important phosphorylated form of α-syn-PS129 involved in PD pathogenesis was not measured in the current study, which should be considered in an expanded cohort in the future.

Earlier observations have shown that erythrocytic α-syn can pass through blood–brain barrier (BBB) *via* extracellular vesicles (EVs) such as exosomes, and intravenous injection of PD mice derived erythrocytic EVs could activate microglia in a pro-inflammatory manner (Matsumoto et al., 2017; Sheng et al., 2020). Considering the large amount of α-syn in erythrocytes, the erythrocytic EV concentrations and the α-syn contained in erythrocytic EVs are promising biomarker candidates for PD diagnosis, which should be investigated next.

## Conclusion

Our current study demonstrated that erythrocytic total and oligomeric α-syn levels are efficient biomarkers for PD diagnosis yielding high sensitivity and specificity. Next, we found a significant correlation of erythrocytic total α-syn concentrations and MDS-UPDRS III scores in PD patients. Furthermore, for the first time we found that erythrocytic total but not oligomeric α-syn levels controlled by age and sex were negatively correlated with anxiety scales assessed by HAMA in PD patients.

## Data Availability Statement

The original contributions presented in the study are included in the article/supplementary material, further inquiries can be directed to the corresponding author/s.

## Ethics Statement

The studies involving human participants were reviewed and approved by the IRB of Beijing Tiantan Hospital, Capital Medical University. The patients/participants provided their written informed consent to participate in this study.

## Author Contributions

ZY and TF designed the study. ZY performed the measurements and wrote the manuscript. GL, EA, and YZ contributed to sample collection and preparation. YL contributed to data analysis. All authors contributed to the article and approved the submitted version.

## Conflict of Interest

The authors declare that the research was conducted in the absence of any commercial or financial relationships that could be construed as a potential conflict of interest. The handling editor JL declared a shared parent affiliation with several of the authors, GL, EA, YZ, and TF, at the time of review.

## Publisher’s Note

All claims expressed in this article are solely those of the authors and do not necessarily represent those of their affiliated organizations, or those of the publisher, the editors and the reviewers. Any product that may be evaluated in this article, or claim that may be made by its manufacturer, is not guaranteed or endorsed by the publisher.

## References

[B1] Barbour R. Kling K. Anderson J. P. Banducci K. Cole T. Diep L. (2008). Red blood cells are the major source of alpha-synuclein in blood. *Neurodegener Dis.* 5 55–59. 10.1159/000112832 18182779

[B2] Broen M. P. Narayen N. E. Kuijf M. L. Dissanayaka N. N. Leentjens A. F. (2016). Prevalence of anxiety in Parkinson’s disease: a systematic review and meta-analysis. *Mov. Disord.* 31 1125–1133. 10.1002/mds.26643 27125963

[B3] Bruno D. Reichert Plaska C. Clark D. P. A. Zetterberg H. Blennow K. Verbeek M. M. (2021). CSF alpha-synuclein correlates with CSF neurogranin in late-life depression. *Int. J. Neurosci.* 131 357–361. 10.1080/00207454.2020.1744596 32228205 PMC7529713

[B4] Caudal D. Alvarsson A. Bjorklund A. Svenningsson P. (2015). Depressive-like phenotype induced by AAV-mediated overexpression of human alpha-synuclein in midbrain dopaminergic neurons. *Exp. Neurol.* 273 243–252. 10.1016/j.expneurol.2015.09.002 26363495

[B5] Cui S. S. Du J. J. Fu R. Lin Y. Q. Huang P. He Y. C. (2017). Prevalence and risk factors for depression and anxiety in Chinese patients with Parkinson disease. *BMC Geriatr.* 17:270. 10.1186/s12877-017-0666-2 29166864 PMC5700465

[B6] El-Agnaf O. M. Salem S. A. Paleologou K. E. Curran M. D. Gibson M. J. Court J. A. (2006). Detection of oligomeric forms of alpha-synuclein protein in human plasma as a potential biomarker for Parkinson’s disease. *FASEB J.* 20 419–425. 10.1096/fj.03-1449com 16507759

[B7] Eusebi P. Giannandrea D. Biscetti L. Abraha I. Chiasserini D. Orso M. (2017). Diagnostic utility of cerebrospinal fluid alpha-synuclein in Parkinson’s disease: a systematic review and meta-analysis. *Mov. Disord.* 32 1389–1400. 10.1002/mds.27110 28880418

[B8] Fan J. Y. Chang B. L. Wu Y. R. (2016). Relationships among Depression, Anxiety, Sleep, and Quality of Life in Patients with Parkinson’s Disease in Taiwan. *Parkinsons Dis.* 2016:4040185. 10.1155/2016/4040185 27293956 PMC4884599

[B9] Fanciulli A. Stankovic I. Krismer F. Seppi K. Levin J. Wenning G. K. (2019). Multiple system atrophy. *Int. Rev. Neurobiol.* 149 137–192. 31779811 10.1016/bs.irn.2019.10.004

[B10] Hong Z. Shi M. Chung K. A. Quinn J. F. Peskind E. R. Galasko D. (2010). DJ-1 and alpha-synuclein in human cerebrospinal fluid as biomarkers of Parkinson’s disease. *Brain* 133 713–726. 10.1093/brain/awq008 20157014 PMC2842513

[B11] Kalia L. V. Lang A. E. (2015). Parkinson’s disease. *Lancet* 386 896–912. 25904081 10.1016/S0140-6736(14)61393-3

[B12] Kano O. Ikeda K. Cridebring D. Takazawa T. Yoshii Y. Iwasaki Y. (2011). Neurobiology of depression and anxiety in Parkinson’s disease. *Parkinsons Dis.* 2011:143547. 10.4061/2011/143547 21687804 PMC3109308

[B13] Maillet A. Krack P. Lhommee E. Metereau E. Klinger H. Favre E. (2016). The prominent role of serotonergic degeneration in apathy, anxiety and depression in de novo Parkinson’s disease. *Brain* 139 2486–2502. 10.1093/brain/aww162 27538418

[B14] Majbour N. K. Vaikath N. N. van Dijk K. D. Ardah M. T. Varghese S. Vesterager L. B. (2016). Oligomeric and phosphorylated alpha-synuclein as potential CSF biomarkers for Parkinson’s disease. *Mol. Neurodegener* 11:7. 10.1186/s13024-016-0072-9 26782965 PMC4717559

[B15] Matsumoto J. Stewart T. Sheng L. Li N. Bullock K. Song N. (2017). Transmission of alpha-synuclein-containing erythrocyte-derived extracellular vesicles across the blood-brain barrier *via* adsorptive mediated transcytosis: another mechanism for initiation and progression of Parkinson’s disease? *Acta Neuropathol. Commun.* 5:71. 10.1186/s40478-017-0470-4 28903781 PMC5598000

[B16] Mollenhauer B. Cullen V. Kahn I. Krastins B. Outeiro T. F. Pepivani I. (2008). Direct quantification of CSF alpha-synuclein by ELISA and first cross-sectional study in patients with neurodegeneration. *Exp. Neurol.* 213 315–325. 10.1016/j.expneurol.2008.06.004 18625222

[B17] Negre-Pages L. Grandjean H. Lapeyre-Mestre M. Montastruc J. L. Fourrier A. Lepine J. P. (2010). Anxious and depressive symptoms in Parkinson’s disease: the French cross-sectionnal DoPaMiP study. *Mov. Disord.* 25 157–166. 10.1002/mds.22760 19950403

[B18] Parnetti L. Gaetani L. Eusebi P. Paciotti S. Hansson O. El-Agnaf O. (2019). CSF and blood biomarkers for Parkinson’s disease. *Lancet Neurol.* 18 573–586. 10.1016/S1474-4422(19)30024-9 30981640

[B19] Piredda R. Desmarais P. Masellis M. Gasca-Salas C. (2020). Cognitive and psychiatric symptoms in genetically determined Parkinson’s disease: a systematic review. *Eur. J. Neurol.* 27 229–234. 10.1111/ene.14115 31686421

[B20] Postuma R. B. Berg D. Stern M. Poewe W. Olanow C. W. Oertel W. (2015). MDS clinical diagnostic criteria for Parkinson’s disease. *Mov. Disord.* 30 1591–1601. 10.1002/mds.26424 26474316

[B21] Pretorius E. Swanepoel A. C. Buys A. V. Vermeulen N. Duim W. Kell D. B. (2014). Eryptosis as a marker of Parkinson’s disease. *Aging* 6 788–819. 10.18632/aging.100695 25411230 PMC4247384

[B22] Reijnders J. S. Ehrt U. Weber W. E. Aarsland D. Leentjens A. F. (2008). A systematic review of prevalence studies of depression in Parkinson’s disease. *Mov. Disord.* 23 183–189. 10.1002/mds.21803 17987654

[B23] Schrag A. (2006). Quality of life and depression in Parkinson’s disease. *J. Neurol. Sci.* 248 151–157. 10.1016/j.jns.2006.05.030 16797028

[B24] Sheng L. Stewart T. Yang D. Thorland E. Soltys D. Aro P. (2020). Erythrocytic alpha-synuclein contained in microvesicles regulates astrocytic glutamate homeostasis: a new perspective on Parkinson’s disease pathogenesis. *Acta Neuropathol. Commun.* 8:102. 10.1186/s40478-020-00983-w 32641150 PMC7346449

[B25] Shi M. Bradner J. Hancock A. M. Chung K. A. Quinn J. F. Peskind E. R. (2011). Cerebrospinal fluid biomarkers for Parkinson disease diagnosis and progression. *Ann. Neurol.* 69 570–580. 10.1002/ana.22311 21400565 PMC3117674

[B26] Shi M. Zabetian C. P. Hancock A. M. Ginghina C. Hong Z. Yearout D. (2010). Significance and confounders of peripheral DJ-1 and alpha-synuclein in Parkinson’s disease. *Neurosci. Lett.* 480 78–82. 10.1016/j.neulet.2010.06.009 20540987 PMC2943649

[B27] Tian C. Liu G. Gao L. Soltys D. Pan C. Stewart T. (2019). Erythrocytic alpha-Synuclein as a potential biomarker for Parkinson’s disease. *Transl. Neurodegener* 8:15. 10.1186/s40035-019-0155-y 31123587 PMC6521422

[B28] Tokuda T. Qureshi M. M. Ardah M. T. Varghese S. Shehab S. A. Kasai T. (2010). Detection of elevated levels of alpha-synuclein oligomers in CSF from patients with Parkinson disease. *Neurology* 75 1766–1772. 10.1212/wnl.0b013e3181fd613b 20962290

[B29] Upneja A. Paul B. S. Jain D. Choudhary R. Paul G. (2021). Anxiety in Parkinson’s Disease: correlation with Depression and Quality of Life. *J. Neurosci. Rural Pract.* 12 323–328. 10.1055/s-0041-1722840 33986584 PMC8110433

[B30] Wang X. Yu S. Li F. Feng T. (2015). Detection of alpha-synuclein oligomers in red blood cells as a potential biomarker of Parkinson’s disease. *Neurosci. Lett.* 599 115–119. 10.1016/j.neulet.2015.05.030 25998655

